# Oral Myiasis in an Immunocompromised Adult Undergoing Chemotherapy: A Rare Case and Comprehensive Treatment Protocol

**DOI:** 10.7759/cureus.42555

**Published:** 2023-07-27

**Authors:** Tarun Kumar Singh, Hariram Sankar, Arshad E, Aakash Gupta, Manish Kumar

**Affiliations:** 1 Department of Dentistry, All India Institute of Medical Sciences, Bathinda, Bathinda, IND; 2 Department of Dentistry, Government Medical College, Ratlam, Ratlam, IND

**Keywords:** oral hygiene, oral cavity, dipteran larvae, parasitic infestation, oral myiasis

## Abstract

Oral myiasis, a rare condition caused by the infestation of live maggots in the oral cavity, can present unique challenges in immunocompromised individuals. This case report presents a unique case of oral myiasis in an immunocompromised adult undergoing chemotherapy. A 67-year-old female suffering from metastatic carcinoma of the ovary was undergoing chemotherapy treatment at the time of presentation. Prompt diagnosis and management, including larval removal, wound care, and systemic antibiotics, were initiated. This case highlights the susceptibility of immunocompromised individuals to uncommon complications, such as oral myiasis, due to their compromised immune system. It also emphasizes the need for heightened vigilance in the oral care and monitoring of immunocompromised patients undergoing chemotherapy, as well as the importance of early intervention to prevent potential complications associated with oral myiasis. In this article, we have also included a comprehensive treatment protocol for treating this condition based on our clinical experience.

## Introduction

Various parasitic infections affect the human race, such as myiasis, which is a particularly devastating infestation caused by common flies. The term myiasis was coined by Hope in 1940, which refers to the invasion of tissues, organs, and certain body cavities of live human vertebrate species by diphtheria eggs or larvae, and commonly manifests as furunculosis lesions [1]. This condition is most commonly reported in tropical and subtropical regions with predominantly warm and humid climates [2]. Infestations are common in various regions of the body such as the skin, nose, eyes, lungs, ears, anus, and vagina. Oral manifestation of the condition is a rare phenomenon. Oral myiasis is associated with various predisposing factors such as mouth-breathing, anterior open bite, incompetent lips (i.e., oral cavity exposed to the external environment), post-tooth extraction, poor oral hygiene, post-maxillofacial fractures, oral carcinoma, and ventilator-supported patients [3]. This condition can occur in both immunocompromised and immunocompetent individuals. It is also common among people living in places/surroundings that lack proper sanitation. In this case report, we present a case of an immunocompromised adult undergoing chemotherapy with oral myiasis.

## Case presentation

**Figure 1:**
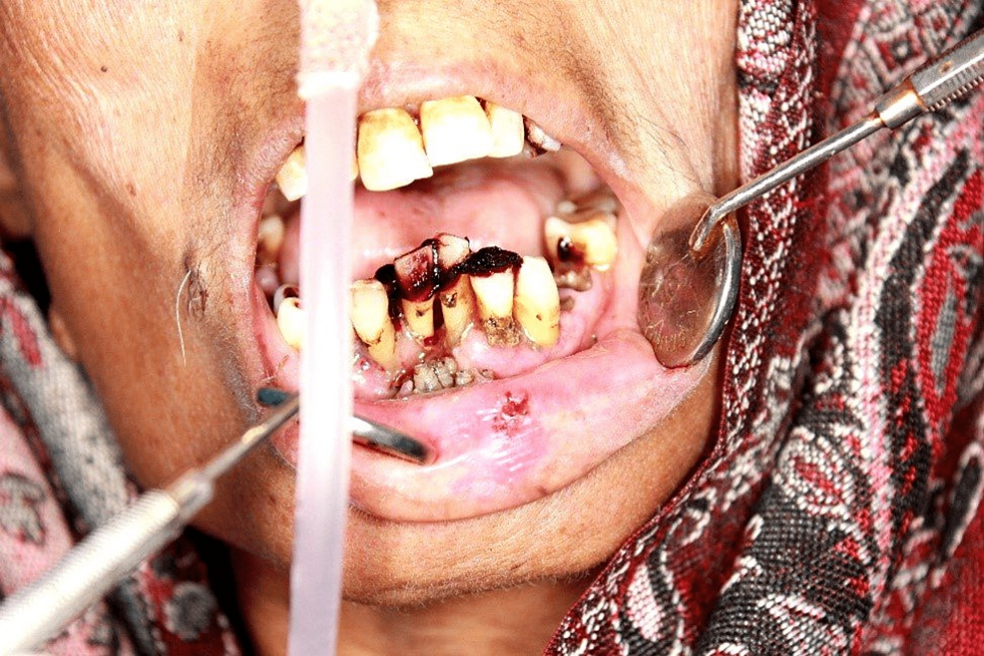
Oral myiasis in a patient with poor oral hygiene.

**Figure 2:**
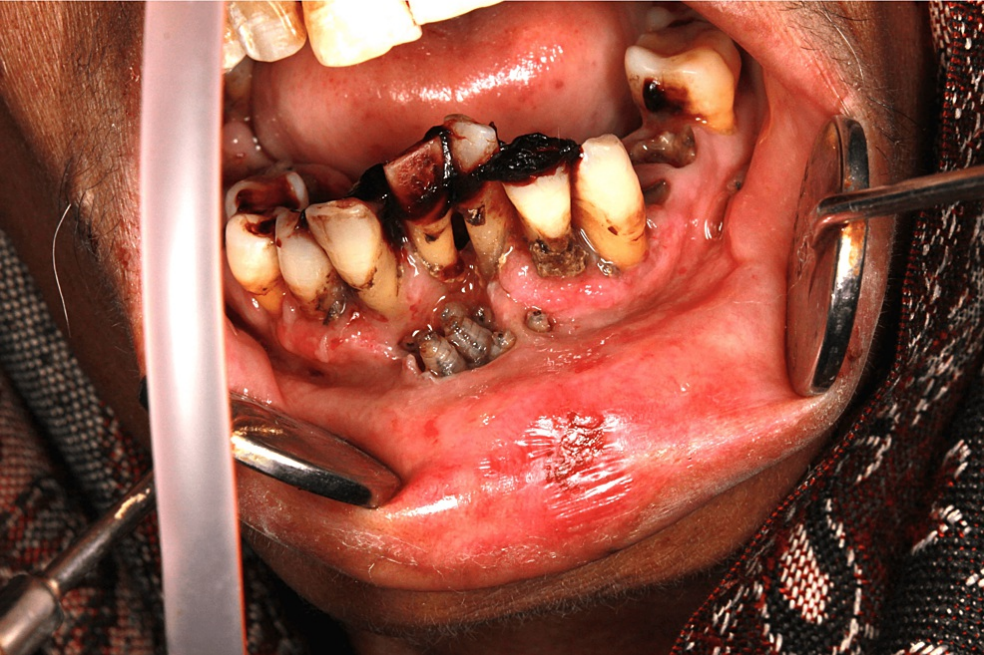
Presence of maggots inside a cavity in the gingivolabial sulcus.

A 67-year-old female suffering from metastatic carcinoma of the ovary for the past nine months reported to our institute. The patient was admitted to the Department of Radiation Oncology for palliative chemotherapy. The patient had already undergone three cycles of chemotherapy. The patient was poorly built, had recent weight loss, and had generalized weakness. Her white blood cell level was low. On the third day of admission, the patient had pain in the lower anterior teeth region and mild swelling of the lower lips. The patient was referred to the Department of Dentistry. On examination, the patient had multiple maggots in the region of the floor of the mouth and anterior gingivolabial sulcus of the oral cavity along with poor oral hygiene, inflamed gingiva, fetid odor, and mobility of lower anterior teeth. On examination with a probe, deep cavitated areas were found in the floor of the mouth and the gingivolabial sulcus with an infestation of a large number of maggots, which required immediate treatment to avoid devastating tissue damage by the larvae (Figures 1, 2).

**Figure 3:**
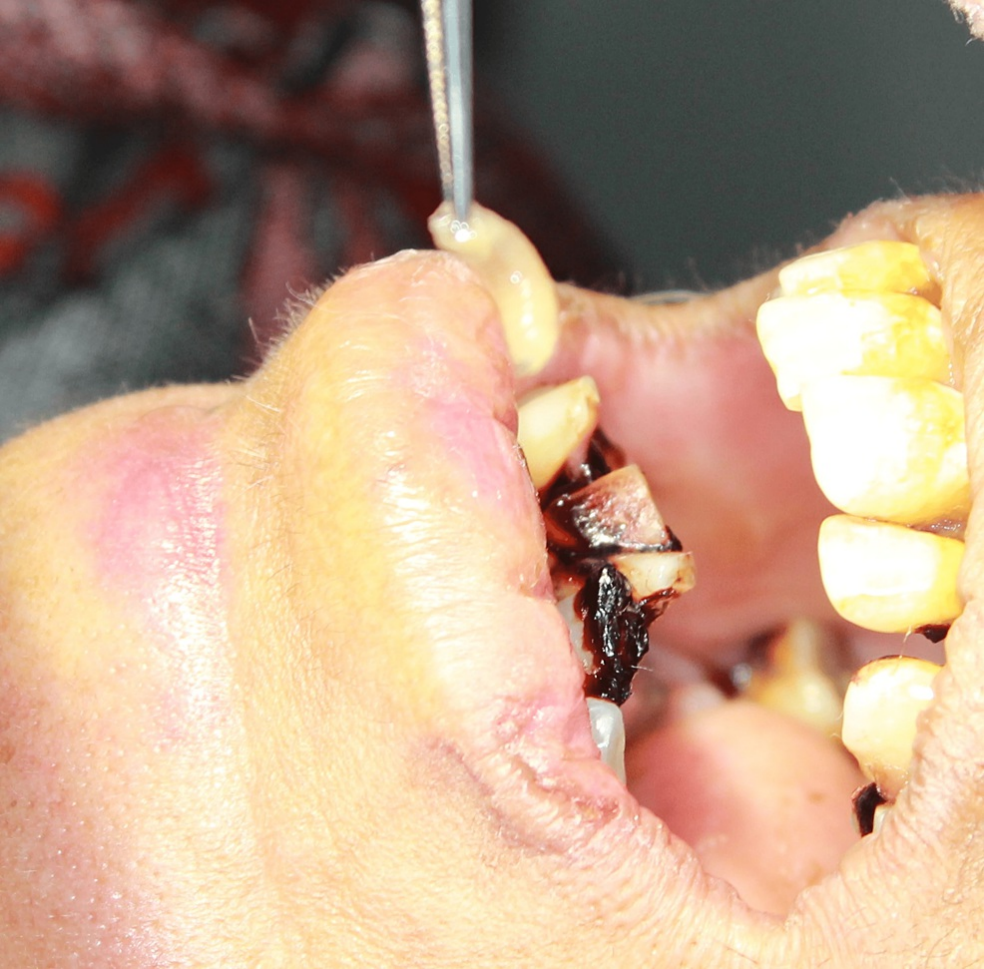
Maggot mechanically removed with a tweezer.

**Figure 4:**
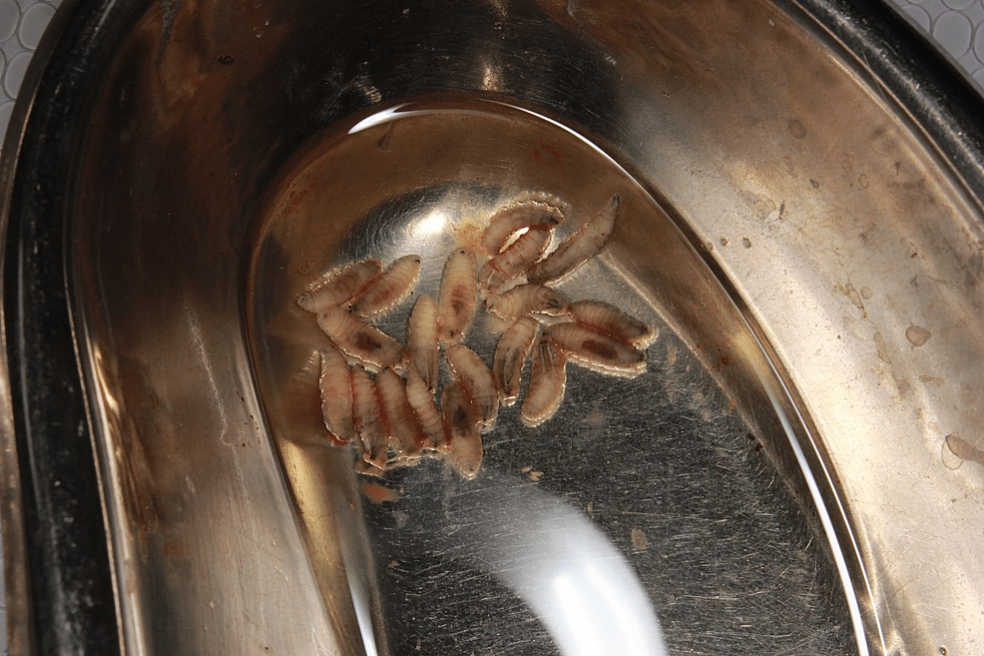
Maggots after removal in a kidney tray.

The treatment involved a controlled local application of turpentine oil soaked in cotton pellets for a few seconds along the cavity, which induced the maggots to come out of the cavities. The maggots were removed mechanically with the help of a dental tweezer, followed by thorough irrigation with saline and povidone-iodine solution. In the first sitting, approximately 70 maggots from the floor of the mouth and 20 maggots from the anterior gingiva-labial sulcus were mechanically removed, after which the patient was relieved of pain. On the second day, the same process was repeated, and 10 to 15 maggots were removed from the same site and thoroughly irrigated (Figures 3, 4).

**Figure 5:**
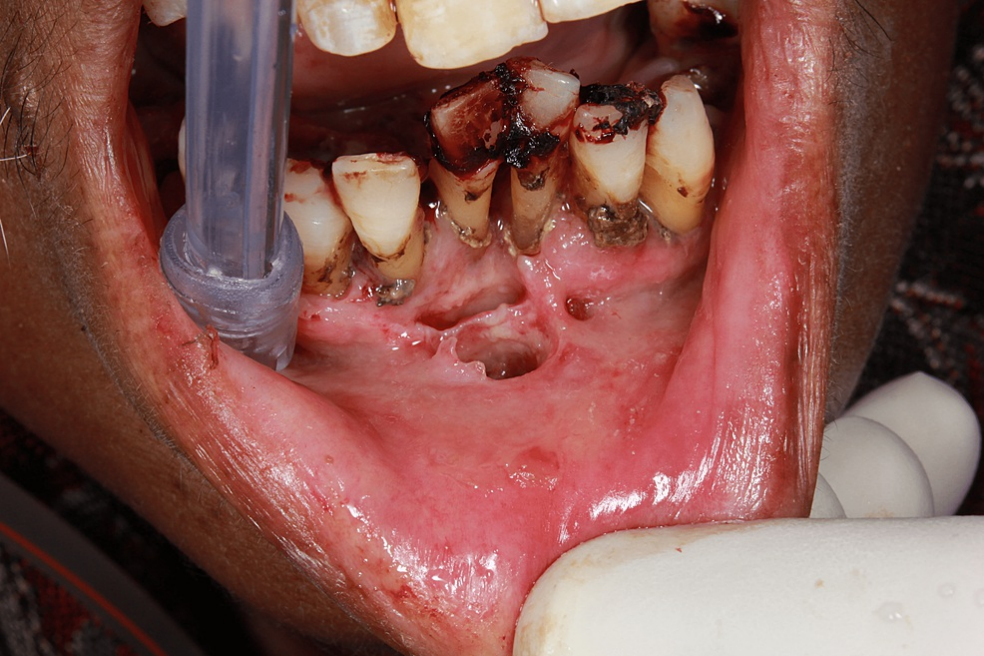
Visibly empty cavity after complete removal of larvae.

On the third day, the patient was completely free of maggot infestation. When re-evaluated after a week, considerable soft tissue healing was found (Figure 5). The patient was simultaneously treated with tablet oral ivermectin 6 mg once daily for three days, tablet metronidazole 400 mg thrice daily for five days, and chlorhexidine mouth rinse for 10 days.

We have developed a comprehensive treatment protocol for patients with oral myiasis. The initial visit involves the following steps: small cotton pellets are soaked with turpentine oil and applied to the affected cavity, which helps to immobilize and detach the maggots from the oral tissues. If necessary, a local anesthetic spray is administered to alleviate pain and discomfort during the procedure. Then, the maggots are mechanically removed from the oral cavity using appropriate instruments. Care is taken to ensure complete removal of all larvae. After maggot removal, the wound and surrounding areas are thoroughly irrigated with a suitable antiseptic solution to eliminate any remaining larvae or debris. The patient is then prescribed oral ivermectin at a dose of 6 mg once daily, which is an effective antiparasitic medication that helps eradicate the larvae. Additionally, oral metronidazole at a dose of 400 mg thrice daily may be prescribed to prevent secondary infections. The patient is advised to use a chlorhexidine mouth rinse to promote oral hygiene and prevent further infections.

On the second and third visits, which occur on consecutive days, the same procedure is repeated to ensure the complete removal of any remaining maggots and to monitor the progress of the healing process. On the fourth visit, scheduled one week later, the patient’s condition is re-evaluated to assess the effectiveness of the treatment and make any necessary adjustments to the treatment plan.

By following the above-mentioned treatment protocol and maintaining close collaboration among healthcare professionals, we can ensure the timely and effective management of oral myiasis cases. This approach aims to achieve optimal outcomes for affected patients while minimizing discomfort, preventing complications, and promoting complete healing.

## Discussion

Oral myiasis is a rare clinical condition that generally occurs among rural populations living close to cattle. In urban populations, oral myiasis typically occurs only in those with poor oral hygiene and poor sanitation. In this specific case, the patient was an end-stage carcinoma ovary patient with generalized body weakness undergoing palliative chemotherapy. She had poor oral hygiene, generalized plaque deposits, gingival inflammation, and fetid odor from the oral cavity. Lack of proper maintenance of oral hygiene could be the main causative factor in this patient. Hence, regular irrigation of the oral cavity with saline and using chlorhexidine mouth rinse should be advised in patients who are suffering from end-stage carcinoma and having difficulty maintaining self-oral hygiene.

Myiasis occurs due to the development of larvae in the tissue, where the human body acts as an intermediate host in their transition phase. The number of developing larvae depends on the number of eggs present [4]. As some maggots may be missed during the first treatment, their mechanical removal should be performed over multiple attempts. In this case, even after the complete mechanical removal of the maggots during the patient’s first visit, some larvae had developed by the time of the second visit. Hence, we developed a comprehensive treatment protocol [5-12] promoting the stepwise removal of maggots, which also includes a follow-up after one week.

The standard treatment of the condition is the immediate mechanical removal of the maggots [5-10], which can be supported with antiparasitic drugs such as ivermectin 12 mg per day for three days and adjuvant clindamycin 300 mg three times per day for five days in cases with severe tissue defects or cavitated lesions involving a large number of larvae. Local application of turpentine oil should be used in minimal quantities in a controlled manner via small cotton pellets [8], as this treatment can cause tissue irritation to the patient if used in large quantities and should be avoided in patients with poor swallow reflexes. Thorough irrigation with normal saline promotes the clearance of any remnant turpentine oil. Many agents can be used for the removal of maggots, such as oil of turpentine, mineral oil, ether, chloroform, ethyl chloride, mercuric chloride, creosote, saline, phenol, calomel, olive oil, iodoform applied locally followed by manual removal, or surgical debridement [11]. In severe cases, even a triple-drug regimen of tablet ivermectin 12 mg per day for three days, tablet albendazole 400 mg twice per day for three days, and tablet clindamycin 300 mg three times per day for five days could be considered [12]. With proper preventive measures, such as maintaining good oral hygiene and proper sanitation, this condition can be easily prevented. The patient should be provided with nutritional support through multivitamin tablets and a proper diet chart. In case of severe pain, the patient can be managed with analgesics. In addition, as patients such as ours have an increased risk of developing secondary skin infections, essential antibiotic therapy should also be considered.

## Conclusions

This case report highlights the rare occurrence of oral myiasis in an immunocompromised adult undergoing chemotherapy, emphasizing the susceptibility of such individuals to opportunistic infections. Prompt diagnosis and a multidisciplinary approach involving dental and medical professionals were crucial for successful management. The case underscores the importance of maintaining a high index of suspicion for unusual oral presentations in immunocompromised patients.
